# Incidence of acquired thrombotic thrombocytopenic purpura in Germany: a hospital level study

**DOI:** 10.1186/s13023-019-1240-0

**Published:** 2019-11-15

**Authors:** Wolfgang Miesbach, Jan Menne, Martin Bommer, Ulf Schönermarck, Thorsten Feldkamp, Martin Nitschke, Timm H. Westhoff, Felix S. Seibert, Rainer Woitas, Rui Sousa, Michael Wolf, Stefan Walzer, Björn Schwander

**Affiliations:** 1 Universitätsklinikum Frankfurt, Medizinische Klinik II / Institut für Transfusionsmedizin und Immunhämatologie, Frankfurt, Germany; 2 Medizinische Hochschule Hannover, Klinik für Nieren- und Hochdruckerkrankungen, Hannover, Germany; 3 Alb-Fils Kliniken Göppingen, Klinik für Hämatologie, Onkologie und Infektionskrankheiten, Göppingen, Germany; 4 Klinikum der Universität München - Medizinische Klinik und Poliklinik IV, Nephrologisches Zentrum, München, Germany; 5 Klinik für Innere Medizin IV - Universitätsklinikum Schleswig Holstein, Nieren- und Hochdruckkrankheiten, Kiel, Germany; 6 Medizinische Klinik I - Universitätsklinikum Schleswig Holstein, Nephrologie & Transplantation, Lübeck, Germany; 7 Medizinische Klinik I – Universitätsklinikum Marien Hospital Herne, Ruhr-Universität Bochum, Herne, Germany; 8 Medizinische Klinik und Poliklinik I - Universitätsklinikum Bonn, Bonn, Germany; 9 Ablynx a Sanofi Company, Medical Affairs, Zwijnaarde, Belgium; 10 MArS - Market Access & Pricing Strategy GmbH, Weil am Rhein, Germany; 11 AHEAD GmbH, Agency for Health Economic Assessment and Dissemination, Lörrach, Germany

**Keywords:** Epidemiology, Germany, Incidence, Thrombotic microangiopathy, Thrombotic thrombocytopenic purpura

## Abstract

**Background:**

Acquired thrombotic thrombocytopenic Purpura (aTTP) is a life-threatening ultra-orphan disease with a reported annual incidence between 1.5 and 6.0 cases per million in Europe and mainly affecting otherwise young and healthy adults aged 40 years on average. The goal of this study was to assess the incidence of aTTP in Germany.

**Methods:**

A systematic review was performed to determine the published evidence on the aTTP epidemiology in Germany. To obtain additional evidence on the proportion of aTTP cases within the national Thrombotic Microangiopathy (TMA) population a hospital-level study was performed, using a retrospective data collection approach. Diagnosis of aTTP was confirmed if ADAMTS13 level were < 10% and/or the medical records explicitly mentioned aTTP diagnosis. The aggregated hospital data were then projected to the national level using logistic regression techniques.

**Results:**

The systematic literature search did not provide incidence estimates of aTTP in Germany. Eight centers (≈27% of the top 30 TMA hospitals) delivered data according to a predefined data collection form. On average (year 2014–2016) a total number of 172 aTTP episodes per year was projected (95% confidence interval [95%CI]: 132–212). The majority were newly diagnosed aTTP cases (*n* = 121; 95%CI: 105–129), and 51 were recurrent aTTP cases (95%CI: 27–84). The average annual projected incidence (year 2014–2016) of aTTP episodes was 2.10 per million inhabitants in Germany (95%CI: 1.60–2.58).

**Conclusions:**

The determined annual incidence of newly diagnosed aTTP cases and the overall annual incidence of aTTP episodes in Germany confirm the ultra-orphan character of aTTP. An external validation against international registries (France, UK and USA) shows that our findings are quite comparable with those international incidence rates.

## Background

Acquired thrombotic thrombocytopenic purpura (aTTP) is a potentially life-threatening thrombotic microangiopathy resulting from systemic microvascular thrombosis and leading to profound thrombocytopenia, hemolytic anemia, and organ failure of varying severity. Acquired TTP is caused by a severe deficiency of ADAMTS13 (a disintegrin and metalloproteinase with a thrombospondin type 1 motif, member 13) due to the presence of inhibitory autoantibodies [1]. Decreased ADAMTS13 activity leads to an accumulation of ultra large von Willebrand factor multimers, which bind to platelets and induce platelet aggregation [2].

These microthrombi cause tissue ischemia and organ dysfunction (commonly involving the brain, heart, and kidneys), resulting in early death [3, 4]. The mortality is up to 90% if untreated, [5] and acute aTTP episodes are still associated with a mortality of 10–20% despite immediate and aggressive therapy including plasma exchange and immunosuppressive strategies [6–9]. In addition to the acute risks of aTTP, long-term follow-up of aTTP patients showed an increased risk of mortality and morbidity. The latter include disabling long-term consequences such as cognitive deficits, depression, and arterial hypertension, and a shortened life expectancy [8, 10–12].

aTTP is an ultra-orphan disease with a reported annual incidence between 1.5 and 6.0 cases per million (reported in French [13],US [14, 15] and UK studies [16, 17]) and mainly affecting otherwise young and healthy adults aged 40 years on average [18, 19]. Hence, the objective of our research was to determine aTTP incidence estimates for Germany by combining different scientific approaches.

## Methods

**Fig. 1:**
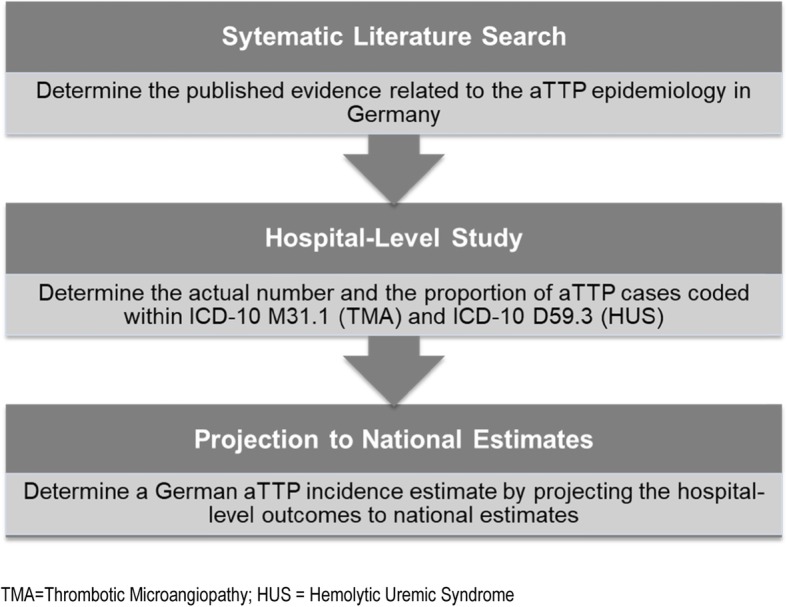
Overview of the main research steps.TMA = Thrombotic Microangiopathy; HUS = Hemolytic Uremic Syndrome

We used a stepwise model of data collection and analysis, presented in Fig. 1.

### Systematic literature search

A systematic literature search, following the PRISMA (Preferred Reporting Items for Systematic Reviews and Meta-Analyses) guidelines, [20] was performed to determine the available published German evidence on the aTTP epidemiology. Details on the methodology of this systematic literature are provided as supporting information in the Additional file 1 of this manuscript.

### Background on national estimates

Acquired TTP and hemolytic uremic syndrome (HUS) belong to a group of diseases known as thrombotic microangiopathies (TMAs), which present with platelet consumption, microangiopathic hemolytic anemia (MAHA) and organ dysfunction resulting from endothelial damage and microvascular thrombosis [21]. Whereas aTTP is primarily associated with central nervous system involvement and HUS is mainly seen as a disease of the kidneys, both are multi-organ diseases and may present with a quite comparable symptom complex and clinical picture, with ADAMTS13 activity as only reliable differentiation criteria [22]. Furthermore in Germany both aTTP and HUS are grouped in the same Diagnosis Related Group (DRG: L72Z named Thrombotic Microangiopathy and Hemolytic Uremic Syndrome) [23]. In Germany the tenth revision of the international classification of diseases (ICD-10) is usually used for coding purposes. As aTTP could either be coded as TMA (ICD-10 M31.1) or HUS (D59.3) national estimates on the annual frequency of both diagnoses (TMA and HUS) were determined from national hospitalization statistics [24–26], and from the German DRG (G-DRG) browser [27–29].

#### National hospitalization statistics

The German Federal Statistical Office provides information on the number of hospitalizations by four-digit ICD-10 main diagnosis [24–26]; there is a duty to provide information defined by the regulation on the federal statistics for hospitals, hence the hospital statistics are an annual total collection of hospitalization cases. The diagnosis data are defined and provided by the hospitals and are based on the main diagnosis at date of discharge. Data from the national hospitalization statistics were used to determine the number of cases with the primary diagnosis related to TMA (M31.1) and HUS (D59.3) per year

#### German diagnosis related group (G-DRG) database

The Institute for the Hospital Remuneration System (InEK; German: Institut für das Entgeltsystem im Krankenhaus) provides DRG codes and hospitalization data based on the G-DRG data delivery, according to section 21 (3) of the Hospital Remuneration Act (German: Krankenhausentgeltgesetz) [27–29]. In contrast to the national hospitalization statistics data provided by the InEK are only reflecting a subset of German hospitals (≈77% 1500 of 1951 hospitals for the year 2016). These data were used to determine the proportion between primary diagnosis and secondary diagnosis related to TMA (M31.1) and HUS (D59.3) per year in Germany. This proportion was then applied to the number of cases with the primary diagnosis of TMA and HUS, determined by the national hospitalization statistics, in order to estimate the number of secondary diagnosis cases for TMA and HUS in Germany

### Hospital-level study

A hospital-level study was performed to determine the proportion of aTTP cases within a population of hospitalized TMA/HUS patients. To figure out the actual number and the proportion of aTTP cases coded within ICD-10 M31.1 (TMA) and ICD-10 D59.3 (HUS), a retrospective epidemiological data collection in Germany hospitals was performed. Based on the ICD-10 code descriptions, it was expected that most of the aTTP cases would be grouped within ICD M31.1 (named “Thrombotic Microangiopathy” including “Thrombotic thrombocytopenic purpura”). In order to identify the German key TMA hospitals, data from the quality reports of the hospitals (German: Qualitätsberichte der Krankenhäuser) [30], were determined as these include the number of TMA hospitalization cases (ICD-10 M31.1) on the hospital level. Using these data, the 30 German hospitals with the highest number of TMA diagnoses in 2013, 2014 and 2015 were determined, (this period refers to data used for hospital selection only) as TMA patients are usually referred to the centers of maximum care that are capable to provide all necessary TMA diagnostics and therapies. These 30 hospitals reflect mainly university hospitals and those were invited to participate in the study. Following the approval by the local ethic committees, the participating hospitals were requested to identify all hospitalization cases with the primary or secondary diagnosis of TMA (M31.1) or HUS (D59.3) recorded between 2014 and 2017, and to extract the following predefined information for each case:

- creation of a patient ID (simple consecutive Arabic case numbers in order to fulfill the requirements of anonymization and pseudonymization)
- 4-digit ICD-10 main diagnosis at the time of hospital discharge (e.g., M31.1)
- 4-digit ICD-10 secondary diagnosis at the time of discharge (only if M31.1 / D59.3)
- month and year of discharge from hospital
- ADAMTS13 activity test was performed (yes / no)
  - If yes, addition of “ADAMTS13 activity in%” (for example, 7.3%)
  - ADAMTS13 auto-antibody test was performed (yes / no)
    - If “yes” the term “positive” / “negative” was added
  - Existence of a known familial disposition for TTP? (Yes / No)
  - further valuable information on the case (e.g. physician rating on aTTP or clarification why no ADAMTS13 test was performed, e.g. recurrent TTP or comparable information).

Patients were considered to have a confirmed diagnosis of aTTP if ADAMTS13 activity levels were < 10% and/or the medical records explicitly mentioned the diagnosis of aTTP. The differentiation between initial and recurrent aTTP episodes was performed on the basis of medical records. By applying this aTTP definition, a conservative approach was chosen as other potential approaches (e.g., ADAMTS13 activity levels < 10% as only criteria) would potentially have led to an underestimation of aTTP cases.

On the basis of these data, the number and proportions of aTTP cases within the ICD-10 M31.1 and the D59.3 (primary and secondary diagnoses at the hospital level) were determined.

### Projection of hospital study outcomes to national estimates

Finally, the proportion determined in the hospital-level study was projected to the national level to elucidate an incidence of aTTP for Germany. On the basis of the outcomes of the hospital-level study, we calculated the proportion of patients with a confirmed diagnosis of aTTP for the primary and secondary diagnosis of TMA and HUS, respectively. In order to reflect the statistical uncertainty related to the hospital study sample a logistic regression analysis, in consideration of the single hospitals as a random effect measure (random effect model), was performed to reflect the 95% confidence intervals (95%CI) around the mean estimates of the related proportions. These proportions and the related 95% CIs were then applied to the national cases for TMA/HUS to project a national estimate of aTTP in Germany. Thereafter, we transferred results into an overall aTTP incidence estimate including both first and recurrent episodes of aTTP.

The determined number of incident aTTP cases are also expressed as cases per million using the number of German inhabitants, based on data from the Federal Statistical Office, related to the year investigated. When reporting the incidence of initial (new) aTTP cases the term “incidence of aTTP” is used, whereas for the overall incidence (initial & recurrent) of aTTP cases the term “incidence of aTTP episodes” is used.

## Results

### Systematic literature search

**Fig. 2:**
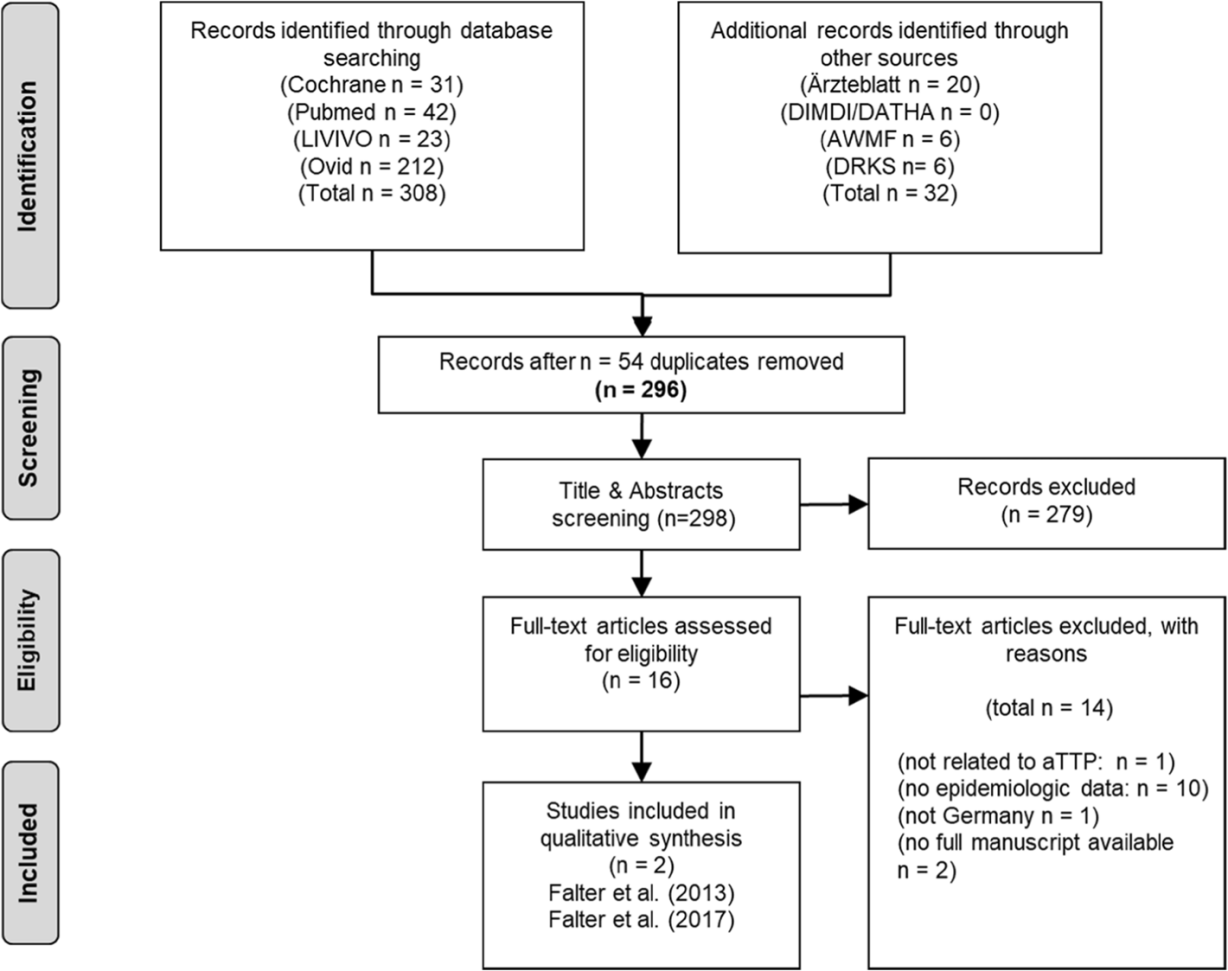
Flow diagram of the systematic review process

In total 340 studies were identified via the database/journal searches, and 296 abstracts were reviewed (database/journal search minus duplicates). From these, 16 articles were selected for full-text review, and 2 papers met the inclusion criteria. The flow chart of study selection is shown in Fig. 2. Detailed information on the systematic literature search strategies’ and outcomes by database / journal are provided in the supporting information (Additional file 1).

As a result of the systematic search we identified two publications [31, 32] that fulfilled all inclusion criteria, but none provided a national estimate on the aTTP incidence or prevalence for Germany. For more information on these studies please refer to the supporting information (Additional file 1).

### National estimates for TMA (M31.1) and HUS (D59.3)

The national estimates for TMA and HUS primary and secondary diagnoses, which are based on the national hospitalization statistics [24–26] and on the G-DRG database [27–29], are presented in Table 1, for the years 2013 to 2016.

**Table 1:** Number of national hospitalization cases for the TMA (M31.1) and HUS (D59.3) according to primary (PD) or secondary diagnosis (SD), year of data collection and patient age

	Year 2016	Year 2015	Year 2014	Year 2013
ICD-10 M31.1 TMA				
M31.1 PD (total)	416	396	407	353
M31.1 PD (18+)	403	381	394	336
M31.1 SD (total)	397	471	352	289
M31.1 SD (18+)	384	453	341	276
ICD-10 D59.3 HUS				
D59.3 PD (total)	467	488	431	391
D59.3 PD (18+)	263	295	251	207
D59.3 SD (total)	267	294	273	214
D59.3 SD (18+)	150	178	159	113

ICD-10 M31.1 “Thrombotic Microangiopathy”, ICD-10 D59.3 “Hemolytic Uremic Syndrome”, *PD* Primary Diagnosis, *SD* Secondary Diagnosis (SD), *total* children, adolescents and adults; *18+* adults 18 years and older

### Hospital-level study

Eight centers (≈27% of the top 30 TMA hospitals requested to participate) delivered data according to a predefined collection form. In total, during the time frame of 2014 to 2017 (hospital study data collection period), 600 hospitalization episodes related to the primary (ICDs M31.1 or D59.3 coded as primary diagnosis) or secondary diagnosis (ICDs M31.1 or D59.3 coded as secondary diagnosis) of TMA/HUS were identified and extracted. As these eight centers are major TMA centers, with a high number of TMA cases, the number of cases that were retrospectively recorded (*n* = 600) reflect approximately 10% of all German HUS / TMA cases observed in a comparable 4-year period (total *n* = 5906 year 2013 to year 2016) [24–29]. The aggregated results by hospital over this four-year period are presented in Table 2.

**Table 2:** Number and proportion of aTTP cases related to all TMA (M31.1) and HUS (D59.3) hospitalization cases per participating hospital (2014–2017)

Sum of Cases (2014–2017)	M	HER	HL	BN	F	KI	GP	H	Total
M31.1 PD	30	16	7	12	29	8	23	87	212
aTTP cases (n)	12	5	3	5	18	2	6	28*	79
aTTP cases (%)	40.0%	31.3%	42.9%	41.7%	62.1%	25.0%	26.1%	32.2%	37.3%
M31.1 SD	36	21	9	15	13	28	0	34	156
aTTP cases (n)	2	0	2	1	1	0	0	2*	8
aTTP cases (%)	5.6%	0%	22.2%	6.7%	7.7%	0.0%	0.0%	5.9%	5.1%
D59.3 PD	8	0	23	14	15	8	7	65	140
aTTP cases (n)	0	0	0	0	0	0	0	[1]*	0
aTTP cases (%)	0.0%	0%	0.0%	0.0%	0.0%	0.0%	0.0%	0.0%	0.0%
D59.3 SD	29	1	9	10	29	10	0	4	92
aTTP cases (n)	0	0	0	0	0	0	0	[1]*	0
aTTP cases (%)	0.0%	0%	0.0%	0.0%	0.0%	0.0%	0.0%	0.0%	0.0%
Total cases (n)	103	38	48	51	86	54	30	190	600
aTTP cases (n)	14	5	5	6	19	2	6	30	87
aTTP cases (%)	13.6%	13.2%	10.4%	11.8%	22.1%	3.7%	20.0%	15.8%	14.5%
Total aTTP (n)	14	5	5	6	19	2	6	30	87
Recurrent aTTP (n)	3	1	0	0	5	0	0	17	26
Recurrent aTTP (%)	21.4%	20.0%	0.0%	0.0%	26.3%	0.0%	0.0%	56.7%	29.9%

ICD-10 M31.1 “Thrombotic Microangiopathy”, ICD-10 D59.3 “Hemolytic Uremic Syndrome”, *PD* Primary Diagnosis, *SD* Secondary Diagnosis (SD), *M* Munich = Medical Clinic and Policlinic IV of the Ludwig-Maximilians-University Munich; HER = Herne = Marien Hospital Herne; HL = Lubeck = University Hospital Schleswig-Holstein, Lubeck; BN = Bonn = University Hospital Bonn; F = Frankfurt am Main = University Hospital Frankfurt; KI = Kiel = University Hospital Schleswig-Holstein, Kiel; GP = Göppingen = Alb Fils Kliniken GmbH; H = Hannover = Clinic for Kidney and Hypertension Disorders; *In Hannover, one aTTP patient was coded as D59.3 PD and one as D59.3 SD; this was identified as miscoded by the study physician; In order to adequately consider these patients, one patient each was classified as M31.1 PD and M31.1 SD and thus assigned to the correct ICD-10 coding

### Projection of hospital study outcomes to National Estimates

**Fig. 3:**
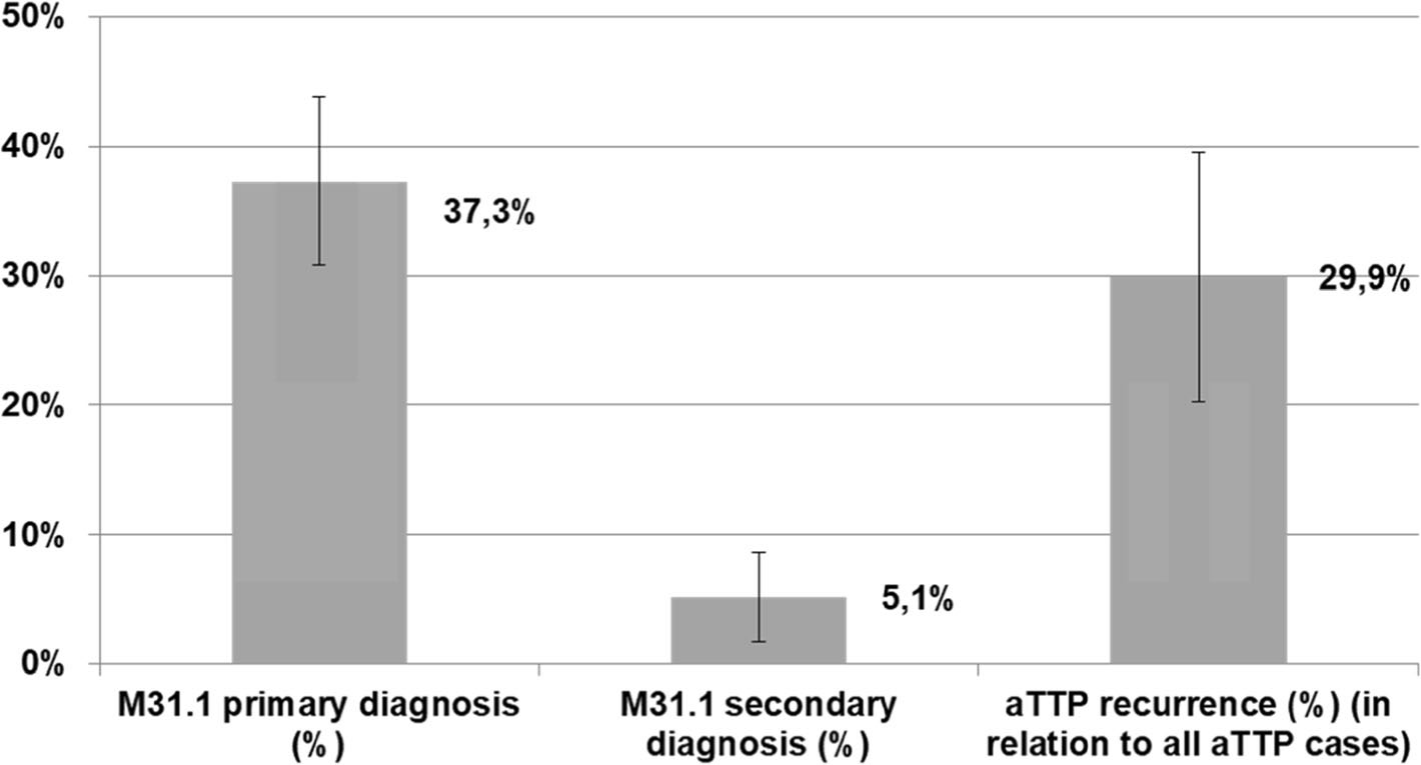
fTYTA Results of the logistic regression: proportion of aTTP cases in patients with a TMA (M31.1) diagnosis based on the hospital-level study outcomes

Using logistic regression analysis, the proportion of patients with a confirmed diagnosis of aTTP was calculated for the primary and secondary diagnosis of TMA (M31.1) on the basis of the outcomes of the hospital-level study. As aTTP cases were only identified in TMA patients (M31.1) the related proportion was computed only for the primary (mean 37.3%; 95%CI: 30.8–43.8%) and secondary TMA (mean 5.1%; 95%CI: 1.7–8.6%) diagnoses. Furthermore the proportion of recurrent aTTP cases (mean 29.9%; 95%CI: 20.3–39.5%) in relation to all aTTP episodes was computed (Fig. 3).

By combining the national estimates (Table 1) and the proportion of aTTP cases, national estimates for acute aTTP episodes were calculated, which are depicted in Table 3.

**Table 3:** Projected annual national acute aTTP cases in the overall population (total) and in adult patients (18+) related to the years 2014 to 2016

Projected national aTTP episodes	Year 2016			Year 2015			Year 2014		
	MV	LL 95%CI	UL 95CI	MV	LL 95%CI	UL 95%CI	MV	LL 95%CI	UL 95%CI
aTTP cases in M31.1 PD (total)	155*	128	182	148	122	173	152	125	178
aTTP cases in M31.1 SD (total)	20	7	34	24	8	40	18	6	30
Overall aTTP cases (total)	175	135	216	172	130	213	170	131	208
aTTP cases (initial manifestation) (total)	123	108	131	121	104	129	119	104	126
aTTP cases (recurrence) (total)	52	27	85	51	26	84	51	27	82
aTTP cases in M31.1 PD (18+)	150	124	176	142	117	167	147	121	172
aTTP cases in M31.1 SD (18+)	20	6	33	23	8	39	17	6	29
Overall aTTP cases (18+)	170	130	209	165	125	206	164	127	201
aTTP cases (initial manifestation) (18+)	119	104	126	116	100	125	115	101	122
aTTP cases (recurrence) (18+)	51	26	83	49	25	81	49	26	79

*example calculation: 37.3% (Fig. 3 M31.1 primary diagnosis (%)) × 416 cases (Table 1 M31.1 PD (total)) = 155.2 cases (rounded = 155 cases), ICD-10 M31.1 “Thrombotic Microangiopathy”, PD = Primary Diagnosis, SD = Secondary Diagnosis (SD); total = children, adolescents and adults; 18+ = adults; MV = mean value, LL 95%CI = lower limit 95% confidence interval; UL 95%CI = upper limit 95% confidence interval

Data are given for the total population (including children, adolescents and adults) and for adults only (18+). The average annual values (year 2014–2016) were 166 (95%CI: 127–205) acute aTTP episodes projected in adults (18+) and 172 (95%CI: 132–212) in the overall population. The majority of acute aTTP cases in the overall population occurred as initial manifestations (average annual values year 2014–2016: 121; 95%CI: 105–129).

Using the number of inhabitants in Germany of the related years (year 2016: total: 82.50 million; 18+: 69.05; year 2015: total: 82.20; 18+:68.80; year 2014: total: 81.20; 18+: 67.96) [33, 34] the number of incident acute aTTP episodes (reported in Table 3) was transferred into cases per million inhabitants (Table 4). In the overall population (adults and children) the average annual incidence (year 2014–2016) of aTTP was 1.47 (95%CI: 1.28–1.57) and the average incidence of aTTP episodes was 2.10 (95%CI: 1.60–2.58).

**Table 4:** Projected annual national incidence estimates for aTTP expressed as cases per million inhabitants in the overall population (total) and in adult patients (18+) related to the years 2014 to 2016

Projected national aTTP episodes (per million)	Year 2016			Year 2015			Year 2014		
	MV	LL 95%CI	UL 95CI	MV	LL 95%CI	UL 95%CI	MV	LL 95%CI	UL 95%CI
aTTP cases per million in M31.1 PD (total)	1.88*	1.55	2.21	1.79	1.47	2.09	1.87	1.54	2.19
aTTP cases per million in M31.1 SD (total)	0.24	0.08	0.41	0.29	0.10	0.48	0.22	0.07	0.37
Overall aTTP cases per million (total)	2.12	1.63	2.62	2.08	1.57	2.57	2.09	1.61	2.56
aTTP cases per million (initial) (total)	1.49	1.31	1.59	1.46	1.26	1.56	1.47	1.28	1.55
aTTP cases per million (recurrence) (total)	0.63	0.32	1.03	0.62	0.31	1.01	0.62	0.33	1.01
aTTP cases per million in M31.1 PD (18+)	2.17	1.80	2.55	2.06	1.70	2.43	2.16	1.78	2.53
aTTP cases per million cases in M31.1 SD (18+)	0.29	0.09	0.48	0.33	0.12	0.57	0.25	0.09	0.43
Overall aTTP cases per million (18+)	2.46	1.89	3.03	2.39	1.82	3.00	2.41	1.87	2.96
aTTP cases per million (initial) (18+)	1.72	1.51	1.82	1.69	1.45	1.82	1.69	1.49	1.80
aTTP cases per million (recurrence) (18+)	0.74	0.38	1.21	0.70	0.37	1.18	0.72	0.38	1.16

* example calculation: 155 cases (Table 3 aTTP cases in M31.1 in 2016) per 82.5 million inhabitants in 2016 = 1.88 acute aTTP cases per million, ICD-10 M31.1 “Thrombotic Microangiopathy”, PD = Primary Diagnosis, SD = Secondary Diagnosis (SD); total = children, adolescents and adults; 18+ = adults; MV = mean value, LL 95%CI = lower limit 95% confidence interval; UL 95%CI = upper limit 95% confidence interval

## Discussion

On average (year 2014–2016) a total number of 172 aTTP episodes per year was projected (95% confidence interval [95%CI]: 132–212) in the German overall population (children and adults). The majority were newly diagnosed aTTP cases (*n* = 121; 95%CI: 105–129), and 51 were recurrent aTTP cases (95%CI: 27–84). The related average annual aTTP incidence was 1.47 per million inhabitants (95%CI: 1.28–1.57) and the related average annual incidence of aTTP episodes was 2.10 per million inhabitants (95%CI: 1.60–2.58). Looking at the German adult population the average annual aTTP incidence was 1.70 per million adults (95%CI: 1.48–1.81) and the related average annual incidence of aTTP episodes was 2.42 per million adults (95%CI: 1.86–3.00). As determined by our systematic literature search these findings represent the first national incidence estimates for aTTP in Germany.

The described projection is bound to the national estimates of TMA (ICD-M31.1) that are based on the national hospitalization statistics and on the G-DRG database [24–29], hence the available national data defined the maximum limit of the projection. Based on the hospital-level data we determined the proportion of confirmed aTTP diagnoses in primary and secondary TMA cases. To account for statistical uncertainty of the study sample, the data from the eight participating hospitals were utilized using logistic regression. This statistical uncertainty was expressed by the lower and upper 95%CI around the presented incidence estimates.

As TMA and HUS are both assigned mainly to the same DRG in Germany (L72Z named Thrombotic Microangiopathy and Hemolytic Uremic Syndrome) [23], we also looked for aTTP patients coded as HUS (ICD-10 D59.3). By these means we identified two aTTP cases that were primarily miscoded as HUS (please refer to Table 2). Therefore, for the projection we considered these cases as TMA. No further aTTP cases were coded as HUS, so for the projection the national data for TMA (ICD-10 M31.1) were used as basis.

In our study, patients were considered to have a confirmed diagnosis of aTTP if ADAMTS13 activity were < 10% and/or the medical records explicitly mentioned the diagnosis of aTTP. A potential alternative definition would have been ADAMTS13 activity < 10% (as only criterion) or ADAMTS13 activity < 10% combined with a positive autoantibody (AAB) test, with the disadvantage that non-tested readmissions and borderline results (rated as aTTP) would have been excluded, both of which might have been resulted in an underestimation of the aTTP incidence.

Hence the applied definition of a confirmed diagnosis of aTTP was rated as the most reliable definition as it may deliver the most realistic estimate and no systematic underestimation on the number of aTTP cases. In 17% of cases (*n* = 15 of *n* = 87 cases) there was no current ADAMTS13 activity measurement available. However, all those cases without ADAMTS13 activity measurement were classified as recurrent aTTP by the treating physician based on the clinical symptoms and by confirmed prior aTTP episodes. In further 11% patients (*n* = 10 of *n* = 87 cases) the ADAMTS13 activity measurements presented borderline values (slightly above 10%) but these were rated as aTTP by the treating physicians (e.g. as the patient had a previous aTTP episode or as ADAMTS13 activity was measured after first plasma exchange therapy). In this context it is also of interest, that only for 59% of all study cases (351 of 600 cases) ADAMTS13 measurements were available, which explains why the ADAMTS13 activity alone was not regarded as reliable definition for the diagnosis of aTTP in the presented study.

The described approach might also include congenital TTP cases as anti-ADAMTS13 antibodies measurement was only available in 69% of patients (*n* = 60 of *n* = 87 cases); hence a differentiation between congenital TTP and acquired TTP, on the basis of the absence of anti-ADAMTS13 antibodies, was not possible in all patients. However, due to the rare event of congenital TTP forms and thorough clinical evaluation the used approach most likely has only a mild impact for overestimation of the aTTP incidence.

For better comparison of different studies detailed analysis of the used methodologies is essential. In this context it is most important to assure that only aTTP was included, whether initial and/or recurrent aTTP episodes were considered, what kind of populations (e.g. adults/children/total) were analyzed and which approach was used for the definition / diagnosis of aTTP (e.g. ADAMTS 13 activity < 10%).

In other countries, the TTP incidence was estimated at 1.5 (France) [13], 3.1 (USA) [14], and 6.0 (UK) [16, 17] cases per million. These varying incidence rates are a result of varying definitions and of population-based differences.

The data from the French registry were calculated on the basis of a large cohort, which was enrolled in the registry over 15 years. They only included patients with a first TMA episode and an ADAMTS13 activity < 10% which was measured in one reference center with a highly standardized method. Therefore the quality of the results can be considered as very reliable. Looking at the applied methods the comparison of our approach to the French cohort seems to be reasonable with the difference that we also report the incidence of recurrent aTTP episodes, while the French group reports only the incidence of (initial) aTTP. Comparing the results we show a good accordance, as in our projection for Germany 1.47 per million inhabitants (1.5 per million in France) are estimated to have an initial aTTP manifestation. In adults the related incidence of aTTP is 1.70 per million.

In the UK TTP registry the incidence of TTP episodes was calculated on the basis of a clinical diagnosis according to national guidelines [35], excluding other conditions such as HUS and HELLP, but patients with secondary TTP (e.g. due to HIV infection or drug-induced TTP) were included [16, 17]. As this approach did not rely on ADAMTS13 measurement, the real incidence of aTTP might have been overestimated in the UK study.

In the United States the Oklahoma TTP-HUS registry provides estimates for the incidence of initial and recurrent aTTP in a mixed population (adult and children), allowing a good comparison to our assessment. Reese et al. reported a standardized aTTP incidence rate (newly diagnosed cases) of 2.17 (95%CI: 2.00–2.34) per million observed in the Oklahoma TTP-HUS registry [14], compared to 1.47 (95%CI: 1.28–1.57)) per million population in our study. Page et al. Identified a combined incidence of initial and recurrent aTTP episodes of 3.1 per million [15] as compared to 2.10 per million inhabitants (95%CI: 1.60–2.58) identified in the present study. The slightly lower incidence found in our study in our study could be explained with the predominant Caucasian origin of the German population in contrast to the Oklahoma registry. As the aTTP incidence was found to be higher in the black population compared to non-blacks (Incidence rate ratio 7.09), [14] this might explain the higher incidence observed in the Oklahoma registry.

Besides the aspects that were already discussed above, there are further limitations of our study. Our incidence estimate is reflecting a mainly Caucasian population and only eight of thirty invited hospitals provided retrospective data and were hence included into this study. Centers from the North, West and South of Germany were equally represented, with a lack of centers from the eastern parts of Germany. As these eight centers are major TMA centers the number of cases that were retrospectively recorded (*n* = 600) reflects approximately 10% of all German HUS / TMA cases (*n* = 5906) [24–29] in this period. With regard to the patient numbers (based on the primary TMA diagnosis) the participating centers reflect about 25% of patients in all top 30 TMA hospitals. However, the logistic regression analysis of the hospital-level data helps to describe potential inaccuracies with 95% confidence intervals.

A further limitation is that we have only selected major TMA centers as basis for our assessment. It is difficult to predict, but possible, that the inclusion of smaller centers (outside the top 30) might have altered the proportion of aTTP cases in relation to all TMA hospitalization cases. However, as our findings compare well to the findings of the French [13] and the Oklahoma [14] aTTP registry, the impact of this potential selection bias is rated minor.

## Conclusions

The determined average annual incidence of newly diagnosed aTTP cases (1.47 per million inhabitants; 1.70 per million adults) and the overall average annual incidence of aTTP episodes (2.10 per million inhabitants and 2.42 per million adults, respectively) in Germany confirm the ultra-orphan character of aTTP. An external validation against international registries (France, UK and USA) shows that our findings are quite comparable with those international incidence rates.

## Additional file

**Additional file 1.** Protocol and results of systematic literature search on German epidemiology data about thrombotic thrombocytopenic purpura (TTP).

## Data Availability

All data generated or analyzed during this study were included in this published article [and its supplementary information files].

## Abbreviations

AAB: Autoantibody
ADAMTS13: A disintegrin and metalloproteinase with a thrombospondin type 1 motif, member 13
aTTP: Acquired thrombotic thrombocytopenic purpura
CI: Confidence interval
DRG: Diagnosis related group
G-DRG: German diagnosis related group
HUS: Hemolytic uremic syndrome
ICD-10: International classification of diseases - tenth revision
InEK: Institute for the hospital remuneration system; German: Institut für das Entgeltsystem im Krankenhaus
MAHAT: Microangiopathic hemolytic anemia and thrombocytopenia
PRISMA: Preferred reporting items for systematic reviews and meta-analyses
TMA: Thrombotic Microangiopathy
