# Supplementary material for: Incidence of acquired thrombotic thrombocytopenic purpura in Germany: a hospital level study
Source: Orphanet J Rare Dis. 2019 Nov 15;14:260. doi: 10.1186/s13023-019-1240-0 (PMC6858672; doi:10.1186/s13023-019-1240-0)
Supplement: Supplementary file 1 — Additional file 1. Protocol and results of systematic literature search on German epidemiology data about thrombotic thrombocytopenic purpura (TTP). [file 13023_2019_1240_MOESM1_ESM.docx]

**Additional file 1**

**Protocol and results of systematic literature search on German epidemiology data about thrombotic thrombocytopenic purpura (TTP)**

The literature search was performed on 22^nd^ July 2018 through application of a “dual control principle”.

**Objectives**

The systematic literature search was supposed to obtain an overview about epidemiological, Germany-specific data on acquired thrombotic thrombocytopenic purpura (aTTP). It was the aim to get data about the incidence and/or prevalence of TTP in Germany.

**Methods**

It was searched for studies that focused on the following three aspects:

- (a)TTP as a primary indication
- Epidemiological data (especially incidence and/or prevalence)
- German population

Studies were included from 1990 onwards due to the reunification of Germany in 1989.

Overview of bibliographic search – Information sources

The bibliographic search included the following **English** databases:

- Cochrane Library <http://cochranelibrary-wiley.com/cochranelibrary/search/>
- PubMed (<https://www.ncbi.nlm.nih.gov/pubmed/>)
- LIVIVO (Leibniz Information Center on life sciences) <https://www.livivo.de/>
- Embase on OVID SP <https://ovidsp-uk-ovid-com>

It was complemented with the following **German** sources:

- Ärzteblatt <https://www.aerzteblatt.de/>
- DIMDI/DAHTA <https://portal.dimdi.de/hta/servlet/Gate?#__DEFANCHOR_>
- AWMF <https://www.awmf.org/leitlinien/leitlinien-suche.html>
- DRKS (German Register Clinical Studies) <https://www.drks.de>

Search strings and keywords – Search strategy

The **English** search strategy for the bibliographic search in **Cochrane, LIVIVO and Medline/PubMed** was set up as follows:

*(Thrombotic microangiopath* OR thrombotic thrombocytopenic* purpura* OR Moschcowitz OR Microangiopath* Hemolytic Anemia OR Schulman Upshaw Syndrome OR Schulman factor OR ICD M31.1) AND (incidence OR prevalence OR morbidity) AND Germany*

In Cochrane, it was additionally searched for *“thrombotic thrombocytopenic purpura AND incidence”* and *“thrombotic thrombocytopenic purpura AND prevalence”*

For **Embase on OVID SP** the following was applied:

*(Thrombotic microangiopathy OR thrombotic thrombocytopenic purpura OR Moschcowitz OR Microangiopathic Hemolytic Anemia OR Schulman Upshaw Syndrome OR Schulman factor OR ICD M31.1) AND (incidence OR prevalence OR morbidity OR epidemiology) AND Germany*

For the German **Ärzteblatt** the following search terms were applied *“Purpura thrombotisch thrombozytopenische”* as well as additionally *“Moschowitz”*

In **DIMDI/DAHTA, AWMF and DRKS** the search terms “Purpura thrombotisch thrombozytopenische” as well as additionally “Moschcowitz” were applied.

**Data management**

The following table gives an overview about the databases, search strings and hits related to the databases and search strings described above.

**Appendix Table 1 – Overview of Search terms and hits (according to G-BA appendix 4a)**

| **Database** | **Search Date** | **Search string** | **Hits** |
| --- | --- | --- | --- |
| Cochrane Library | 22^nd^ July 2018 | #1 thrombotic thrombocytopenic purpura AND incidence | 28 |
|  |  | #2 thrombotic thrombocytopenic purpura AND prevalence | 5 |
|  |  | #3 (Thrombotic microangiopath* OR thrombotic thrombocytopenic* purpura* OR Moschcowitz OR Microangiopath* Hemolytic Anemia OR Schulman Upshaw Syndrome OR Schulman factor OR ICD M31.1) AND (incidence OR prevalence OR morbidity) AND Germany | 1 |
|  |  | **Combined Searches (#1 OR #2 OR #3) excluding duplicates (Search Filter Publication Year from 1990 to 2018)** | **31** |
| PubMed | 22^nd^ July 2018 | (Thrombotic microangiopath* OR thrombotic thrombocytopenic* purpura* OR Moschcowitz OR Microangiopath* Hemolytic Anemia OR Schulman Upshaw Syndrome OR Schulman factor OR ICD M31.1) AND (incidence OR prevalence OR morbidity) AND Germany **(Search Filter Publication Year from 1990 to 2018)** | **42** |
| LIVIVO | 22^nd^ July 2018 | (Thrombotic microangiopath* OR thrombotic thrombocytopenic* purpura* OR Moschcowitz OR Microangiopath* Hemolytic Anemia OR Schulman Upshaw Syndrome OR Schulman factor OR ICD M31.1) AND (incidence OR prevalence OR morbidity) AND Germany **(Search Filter Publication Year from 1990 to 2018)** | **23** |
| Embase on OVID SP | 22^nd^ July 2018 | (Thrombotic microangiopathy OR thrombotic thrombocytopenic purpura OR Moschcowitz OR Microangiopathic Hemolytic Anemia OR Schulman Upshaw Syndrome OR Schulman factor OR ICD M31.1) AND (incidence OR prevalence OR morbidity OR epidemiology) AND Germany**(Search Filter Publication Year from 1990 to 2018)** | **212** |
| Ärzteblatt | 22^nd^ July 2018 | #1 “Purpura thrombotisch thrombozytopenische” **(Search Filter Publication Year from 1990 to 2018)** | 16 |
|  |  | #2 “Moschcowitz” **(Search Filter Publication Year from 1990 to 2018)** | 8 |
|  |  | **Combined Searches (#1 OR #2) excluding duplicates (Search Filter Publication Year from 1990 to 2018)** | **20** |
| DIMDI/DAHTA | 22^nd^ July 2018 | Purpura thrombotisch thrombozytopenische OR Moschcowitz | 0 |
| AWMF | 22^nd^ July 2018 | #1 Purpura thrombotisch thrombozytopenische | 6 |
|  |  | #2 Moschcowitz | 0 |
|  |  | **Combined Searches (#1 OR #2) excluding duplicates (Search Filter Publication Year from 1990 to 2018)** | **6** |
| DRKS (German Register Clinical Studies) | 22^nd^ July 2018 | #1 Purpura thrombotisch thrombozytopenische | 6 |
|  |  | #2 Moschcowitz | 0 |
|  |  | **Combined Searches (#1 OR #2) excluding duplicates (Search Filter Publication Year from 1990 to 2018)** | **6** |
| Overall Results | 22^nd^ July 2018 | Overall results (including duplicates between databases) | 340 |
|  |  | Overall results /excluding duplicates) | 296 |

**Selection process**

The selection process was conducted independently by two researchers as to ensure a “dual control principle” (see Figure 1 in the main manuscript).

14 full text articles were excluded for the following reasons: article not focusing on aTTP (n=1), no epidemiological data (incidence or prevalence) included (n=10), not focusing on a German population (n=1), no full manuscript available (n=2).

The following exclusion criteria (EC) were applied:

- EC 1: aTTP not primary indication
- EC 2: No aTTP-specific epidemiological data provided
- EC 3: No focus on German population
- EC 4: Articles older than 1990
- EC 5: Other language than English or German
- EC 6: Press releases
- EC 7: No full manuscript available (e.g. congress reports / posters)
- EC 8: Notifications on new active substances or pharmaceuticals
- EC 9: Case studies, case reports

During the selection process, the first exclusions were based upon duplicated and the screening of titles and abstracts. The above listed exclusion criteria were applied as far as possible. Whenever unsure about the content of the study, it remained included for full-text screening. All records that were rated as relevant by one of the reviewers were included into the full-text review.

The selection described above was repeated for the full text reviews. Discrepancies within the final full-text selection / inclusion were discussed during the authors. For this discrepancy solution and for each decision at any time of the process, a rather cautious approach was chosen in order to ensure that no potentially relevant record is excluded.

As a result, **two publications** (presented in appendix table 2) were left that fulfilled the three main aspects of the systematic literature review: to obtain an overview about epidemiological, Germany-specific data on thrombotic thrombocytopenic purpura (TTP).

**Appendix Table 2: Key information from selected studies**

| **Author / Year** | **Falter et al. 2017[1]** | **Falter et al. 2013[2]** |
| --- | --- | --- |
| **Title** | Depression and cognitive deficits as long-term consequences of thrombotic thrombocytopenic purpura | Long term outcome and sequelae in patients after acute thrombotic thrombocytopenic purpura episodes |
| **Patient Selection** | Patients were prospectively registered, who had been treated for acute TTP and/or followed in remission at least once per year | Patients were recruited of a collective of TTP patients who have been completely observed from first manifestation until the study was conducted |
| **Description Patient Cohort** | A total of 148 patients with a clinical diagnosis of aTTP, one with a hereditary TTP, and 52 healthy controls were asked to participate | The long term outcome and sequelae of 21 patients from different nationwide clinics with 103 acute clinical episodes of TTP over 30 years was described |
| **n study subjects** | 201 | 21 |
| **n TTP Patients** | 149 | 21 |
| **Incidence TTP** | One center incidence over 18 months: Nineteen patients had their initial diagnosis of TTP during this study | Data on relapse risk: The relapse risk per month for these 21 TTP patients is 0.026. |
| **Prevalence TTP** | One center prevalence: n=148 – but total time horizon is unclear; no information on aTTP recurrence provided |  |

An overview of the excluded full-texts including the resaonbs for the exclusion is provided in appendix table 3 below.

**Appendix Table 3: Excluded full-texts including reason**

| **Study #** | **Author** | **Year** | **Title** | **Reason for Exclusion** |
| --- | --- | --- | --- | --- |
| 1 | John, M et al. | 2012 | Autoimmune disorders in patients with idiopathic thrombotic thrombocytopenic purpura | EC 2: No aTTP-specific epidemiological data (prevalence of co-occurring immunologic disorders in aTTP) |
| 2 | Hollenbeck, M.et al. | 1995 | Haemolytic-uraemic syndrome and thrombotic-thrombocytopenic purpura in adults: clinical findings and prognostic factors for death and end-stage renal disease | EC 2: No aTTP-specific epidemiological data are provided (no differentiation between HUS/TTP and no aTTP incidence/prevalence data presented) |
| 3 | Dierkes, F. et al. | 2012 | Indicators of acute and persistent renal damage in adult thrombotic microangiopathy | EC 2: No aTTP-specific epidemiological data are provided (no differentiation between HUS/TTP and no aTTP incidence/prevalence data presented) |
| 5 | Zeitler H. et al. | 2007 | Treatment of adults with clinically suspected severe thrombotic thrombocytopenic purpura - Experiences of a single centre | EC 2: No aTTP-specific epidemiological data are provided (differentiation between HUS/TTP unclear and no aTTP incidence/prevalence data presented) |
| 6 | Müller, A.et al. | 2011 | Hämolytische Anämien beim Erwachsenen | EC 2: No aTTP-specific epidemiological data are provided (just description of disease no aTTP incidence/prevalence data presented) |
| 7 | Kühne, T. et al. | 2007 | Idiopathische thrombozytopenische Purpura im Kindesalter | EC 1: aTTP not primary indication (focus of the paper is ITP = idiopathic thrombozytopenic purpura (ICDD69.3) |
| 8 | Andersohn, F. et al. | 2004 | Proportion of drug-related serious rare blood dyscrasias: estimates from the Berlin Case-Control Surveillance Study | EC 2: No aTTP-specific epidemiological data are provided (relative frequeny of drug-induced blood dyscrasias no aTTP incidence/prevalence data presented) |
| 9 | Bergmann, F. et al. | 2015 | Differenzialdiagnose der Thrombozytopenie in der Schwangerschaft | EC 2: No aTTP-specific epidemiological data are provided (relative frequeny of pregnancy-induced thrombozytpenias [≈1% aTTP] but no aTTP incidence/prevalence data presented) |
| 10 | Benz, K. et al. | 2009 | Pathological aspects of membranoproliferative glomerulonephritis (MPGN) and haemolytic uraemic syndrome (HUS) / thrombocytic thrombopenic purpura (TTP) | EC 2: No aTTP-specific epidemiological data are provided (differentiation between HUS/TTP unclear and no aTTP incidence/prevalence data presented; only epi data for membranoproliferative glomerulonephritis) |
| 11 | (DRKS entry and Schönermarck et al. Poster 2017 - 9. Jahrestagung DGfN) |  | DRKS - Querschnitts-Untersuchung zu klinischen Symptomen und epidemiologischen Parametern in Patienten mit TMA, differenziert durch Laborparameter (CESAR) | EC 7: No full manuscript available Relative frequency of TTP to other TMAs is presented (14%; 31 of 219 in adults) but no manuscript published and no incidence/prevalence value for aTTP in Germany is estimated. |
| 12 | Benz, K. et al. | 2010 | Thrombotic microangiopathy: new insights | EC 2: No aTTP-specific epidemiological data are provided (desciption of different TMAs but no aTTP incidence/prevalence data presented) |
| 13 | von Auer, C. et al. | 2015 | Current insights into thrombotic microangiopathies: Thrombotic thrombocytopenic purpura and pregnancy | EC 2: No aTTP-specific epidemiological data are provided (role of pregnancy in TTP but no aTTP incidence/prevalence data are presented) |
| 15 | von Auer, C. et al. | 2017 | Initiation of a prospective observational registry for patients with acquired thrombotic thrombocytopenic purpura (TTP) in Germany | EC 7: No full manuscript available (e.g. congress reports / posters): Congress presentation no further info available. |
| 16 | Bommer, M. et al. | 2018 | Differenzialdiagnose und Therapie thrombotischer Mikroangiopathien | EC 3: No focus on German population (US incidence 3.1 / Million per Year is provided) |

1. Falter T, Schmitt V, Herold S, Weyer V, von Auer C, Wagner S, et al. Depression and cognitive deficits as long-term consequences of thrombotic thrombocytopenic purpura. Transfusion. 2017;57(5):1152-62.

2. Falter T, Alber KJ, Scharrer I. Long term outcome and sequelae in patients after acute thrombotic thrombocytopenic purpura episodes. Hamostaseologie. 2013;33(2):113-20.
